# Appraising Unmet Needs and Misinformation Spread About Polycystic Ovary Syndrome in 85,872 YouTube Comments Over 12 Years: Big Data Infodemiology Study

**DOI:** 10.2196/49220

**Published:** 2023-09-11

**Authors:** Kashish Malhotra, Punith Kempegowda

**Affiliations:** 1 Department of Surgery Dayanand Medical College and Hospital Ludhiana India; 2 Institute of Applied Health Research College of Medical and Dental Sciences University of Birmingham Birmingham United Kingdom; 3 Queen Elizabeth Hospital University Hospitals Birmingham NHS Foundation Trust Birmingham United Kingdom

**Keywords:** polycystic ovary syndrome, PCOS, public, YouTube, global health, online trends, global equity, infodemiology, big data, comments, sentiment, network analysis, contextualization, word association, misinformation, endocrinopathy, women, gender, users, treatment, fatigue, pain, motherhood

## Abstract

**Background:**

Polycystic ovary syndrome (PCOS) is the most common endocrinopathy in women, resulting in substantial burden related to metabolic, reproductive, and psychological complications. While attempts have been made to understand the themes and sentiments of the public regarding PCOS at the local and regional levels, no study has explored worldwide views, mainly due to financial and logistical limitations. YouTube is one of the largest sources of health-related information, where many visitors share their views as questions or comments. These can be used as a surrogate to understand the public’s perceptions.

**Objective:**

We analyzed the comments of all videos related to PCOS published on YouTube from May 2011 to April 2023 and identified trends over time in the comments, their context, associated themes, gender-based differences, and underlying sentiments.

**Methods:**

After extracting all the comments using the YouTube application programming interface, we contextually studied the keywords and analyzed gender differences using the Benjamini-Hochberg procedure. We applied a multidimensional approach to analyzing the content via association mining using Mozdeh. We performed network analysis to study associated themes using the Fruchterman-Reingold algorithm and then manually screened the comments for content analysis. The sentiments associated with YouTube comments were analyzed using SentiStrength.

**Results:**

A total of 85,872 comments from 940 PCOS videos on YouTube were extracted. We identified a specific gender for 13,106 comments. Of these, 1506 were matched to male users (11.5%), and 11,601 comments to female users (88.5%). Keywords including diagnosing PCOS, symptoms of PCOS, pills for PCOS (medication), and pregnancy were significantly associated with female users. Keywords such as herbal treatment, natural treatment, curing PCOS, and online searches were significantly associated with male users. The key themes associated with female users were symptoms of PCOS, positive personal experiences (themes such as *helpful* and *love*), negative personal experiences (*fatigue* and *pain*), motherhood (*infertility* and *trying to conceive*), self-diagnosis, and use of professional terminology detailing their journey. The key themes associated with male users were misinformation regarding the “cure” for PCOS, using natural and herbal remedies to cure PCOS, fake testimonies from spammers selling their courses and consultations, finding treatment for PCOS, and sharing perspectives of female family members. The overall average positive sentiment was 1.6651 (95% CI 1.6593-1.6709), and the average negative sentiment was 1.4742 (95% CI 1.4683-1.4802) with a net positive difference of 0.1909.

**Conclusions:**

There may be a disparity in views on PCOS between women and men, with the latter associated with non–evidence-based approaches and misinformation. The improving sentiment noticed with YouTube comments may reflect better health care services. Prioritizing and promoting evidence-based care and disseminating pragmatic online coverage is warranted to improve public sentiment and limit misinformation spread.

## Introduction

Polycystic ovary syndrome (PCOS) is the most common endocrinopathy in women, with a prevalence of 8% to 10% worldwide [1]. It results in a substantial economic burden due to metabolic, reproductive, emotional, and psychological complications [2]. A systematic review of the lived experiences of people with PCOS highlighted the need for better public awareness to limit stigma and build positive social support [3]. Several researchers have explored the opinions of the public at local and regional levels [4-6]. However, we did not find similar research at a worldwide level. A recent study highlighted that the lived experiences of people with PCOS are influenced by ethnicity and birthplace [7]. As the logistics and financial burden of conducting large-scale studies to confirm this worldwide would be heavy, we must consider surrogate methods.

Social media can act as a surrogate for public views and sentiments by providing a forum for people to voice their ideas and participate in debates on various subjects. This provides an opportunity for academics and researchers to learn more about the views and opinions of the public. Infodemiology is the science of distribution and determinants of information in an electronic medium, specifically the internet or in a population, with the ultimate aim of informing public health and public policy [8]. Big data analytics have reformed changing health paradigms with rigorous analytical reviews to provide better clinical solutions [9]. This involves formulating quantitative and qualitative assessments previously too large for conventional software [10]. Infodemiology also helps to critically evaluate resource allocation and misinformation spread online, providing evidence-based recommendations for multisectoral actions to develop legal policies and increase health literacy [11]. Our group explored the recurring themes associated with PCOS on Twitter [12,13]. However, Twitter’s ability to reflect the wider world is constrained by linguistic and cultural obstacles and the local concentration of the platform’s user base resulting in a digital divide. Further, Twitter’s algorithms may amplify prejudices and stifle other viewpoints.

YouTube is an online video-sharing platform and is the second most visited website after Google [14]. As the internet continues to develop as the primary source of health-related information, YouTube has a significant potential to drive health-related conversations, with over 2 billion users monthly [15,16]. Analysis of YouTube’s comments provides an excellent opportunity to understand the public’s perceptions on a global scale. While some studies have analyzed the content and reliability of YouTube videos related to reproductive health and PCOS, the findings were limited by small sample sizes and a lack of focus on a comprehensive analysis of the public’s comments [17,18]. Therefore, we delved into the vast realm of YouTube comments on PCOS videos, meticulously analyzing the keywords, sentiments, prevailing trends, and recurring themes expressed by the public, ultimately unravelling the rich tapestry of opinions surrounding PCOS. As recent studies have shown that people with PCOS are not satisfied with the care received, by examining these trends, we contribute to a better understanding of the dynamics of public interactions and shed light on areas for potential improvement in PCOS care delivery [3]. Our specific objectives were (1) to identify trends over time, study the context of YouTube comments about PCOS and find associated themes, (2) to identify gender-based differences in these YouTube comments, and (3) to identify the underlying sentiments of these YouTube comments.

## Methods

### Data Extraction

This study was conducted in May 2023. All videos published on YouTube with “PCOS” OR “polycystic ovary syndrome” in the title or description from May 2011 to April 2023 were included in the study. The earliest possible data available in the public domain were from May 2011. The YouTube application programming interface was used to extract comments on videos. The extracted comments were analyzed using Mozdeh (University of Wolverhampton). Only the initial comment for each individual was included to prevent any individual’s opinions from overshadowing the results. We included up to 350 recent comments to ensure popular videos with more comments did not sway the overall analysis. A similar method has been used elsewhere [19,20].

### Word Association Detection, Contextualization, and Content Analysis

We analyzed the overall keyword frequency to identify recurring themes in the comments. This was performed after removing keywords related to prepositions or other connecting words. Time series graphing was done to understand overall trends in the YouTube comments. Association-mining comparison analysis was done to study differences and formulate a list of keywords for which there was statistical evidence of gender differences in their use by male versus female commentators. Words were considered gendered (ie, had evidence of gender differences) if they met the following criteria: (1) they appeared in at least two profiles (to rule out typos), (2) they appeared in a higher proportion of comments from that gender than from other genders, and (3) the findings were statistically significant.

A 2 × 2 chi-squared test was used to determine whether the evidence supported that a word was used disproportionately by male or female individuals for each word in the overall gendered word frequency table. The Benjamini-Hochberg procedure [21] was used to control the risk of false positives from running multiple tests. It adjusts the chi-squared threshold to keep the likelihood of drawing at least one false positive conclusion below 5%. After identifying gendered keywords, network analysis was done using the Fruchterman-Reingold algorithm [22,23] to identify the connections with other similar keywords and study associated themes. The disconnected nodes were placed in corners, and the node size was set proportional to the number of comments for the label. Finally, after identifying the associated keywords for each gender, the YouTube comments with each of those keywords were manually read to identify underlying themes and were categorized. The reported analysis highlights major themes, subthemes, and representative quotes.

### Gender Identification

Although YouTube does not track commenters’ gender, certain commenters’ usernames can be used to guess their gender. These data served as a proxy for population gender data. Usernames were divided into numerous parts (using spaces or intercapped compounds) where possible. The gender of the commenter was then determined by matching the first part of the name to one used at least 90% by men or women in the 1990 US census [24]. US census data were chosen because the United States is a cosmopolitan country with people from various regions and ethnicities. The appropriate gender was also ascribed for the terms *Mr*, *Mrs*, *Ms*, and *Miss* [19].

### Sentiment Analysis

The strength of the positive and negative sentiment of each comment was identified with SentiStrength, which is incorporated within the Mozdeh software and uses a lexicon of sentiment terms with linguistic rules [25]. Sentiment analysis was done for each comment and was not specifically targeted at only those comments including *PCOS*, *PCOD*, *polycystic ovary syndrome*, or any other similar term. A score of 1 (not positive) to 5 (extremely positive) and a second independent score of 1 (not negative) to 5 (extremely negative) was given to each text. SentiStrength was chosen due to its ability to analyze negative and positive sentiment independently using a dual method, which is crucial for the objectives of the research; its accuracy on YouTube comments is close to human levels [25-27]. For social science research purposes, lexical software that uses a predefined set of sentiment terms with linguistic rules, such as SentiStrength, is preferred to machine learning because the latter can identify contentious themes as a proxy for sentiment.

The average positive and negative sentiment strength of each group’s comments were determined independently. The conventional normal distribution formula was used for each group to construct a 95% CI. Due to the skewed and discrete nature of the data—as opposed to continuous data—this is an approximation. Due to the possibility of interdependence among comments made on the same YouTube video, the data also violates the statistical independence assumption. Therefore, the CIs should be considered indicative estimations rather than robust numbers. To compensate, the changes in average sentiment were considered significant only when CIs did not overlap. As a slight overlap between 2 associated CIs is consistent with statistically significant differences, our methodology is somewhat conservative [28,29]. Sentiment trends were also studied from 2011 to 2023.

### Ethics Statement

No human or animal participants were involved, and no patient data were collected. Only publicly accessible data were analyzed; no identifiable data are reported in this study.

## Results

### Overview

From 940 videos, 85,872 total comments were fetched and analyzed. We identified a specific gender for 13,106 comments. Of these comments, 1506 were matched to male users (11.5%), and 11,601 to female users (88.5%).

### Trends and Keyword Analysis

**Figure 1:**
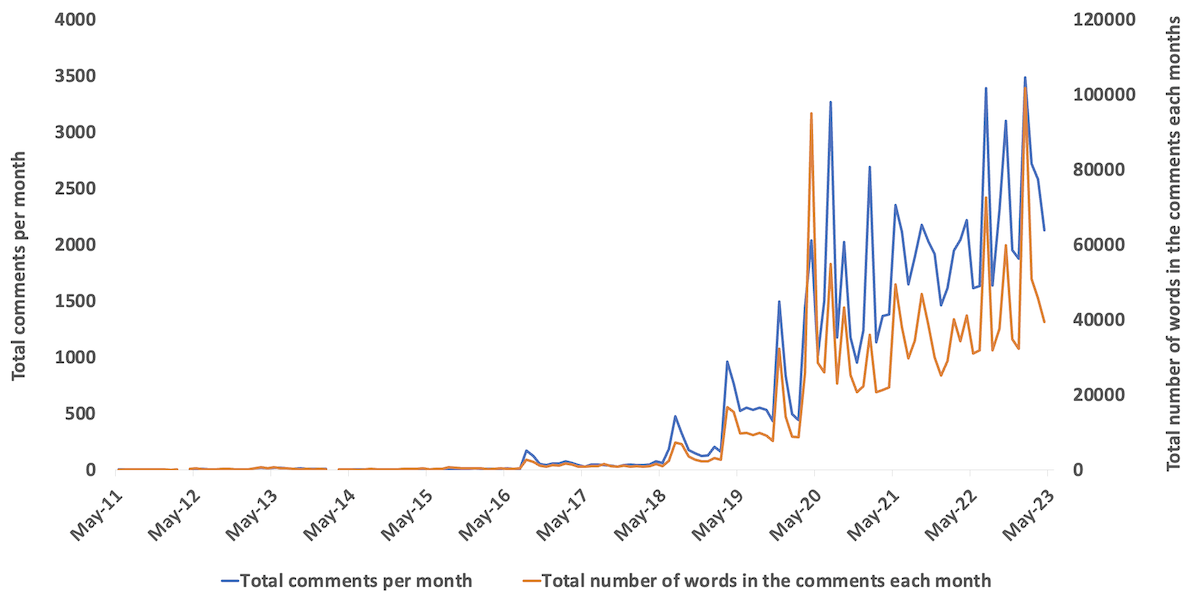
Time series graph of comments on polycystic ovary syndrome videos from May 2011 to April 2023. The primary vertical axis (left) represents total YouTube comments (blue line) and the secondary vertical axis (right) represents total words in YouTube comments (orange line). There were no new comments in March 2012 and February 2014.

We noticed an increasing trend in the total number of comments and the length of YouTube comments by users in the past 5 years (Figure 1). The most frequently used keywords (after excluding words referring to PCOS or the video itself, prepositions, and other connecting words) were *period* (n=9352), *thank* (n=8911), *month* (n=4566), *doctor* (n=4481), *weight* (n=4318), *problem* (n=4204), *help* (n=4086), *hair* (n=3833), *time* (n=3718), and *diet* (n=3262).

### Female Versus Male Word Association, Contextualization, and Content Analysis

**Figure 2:**
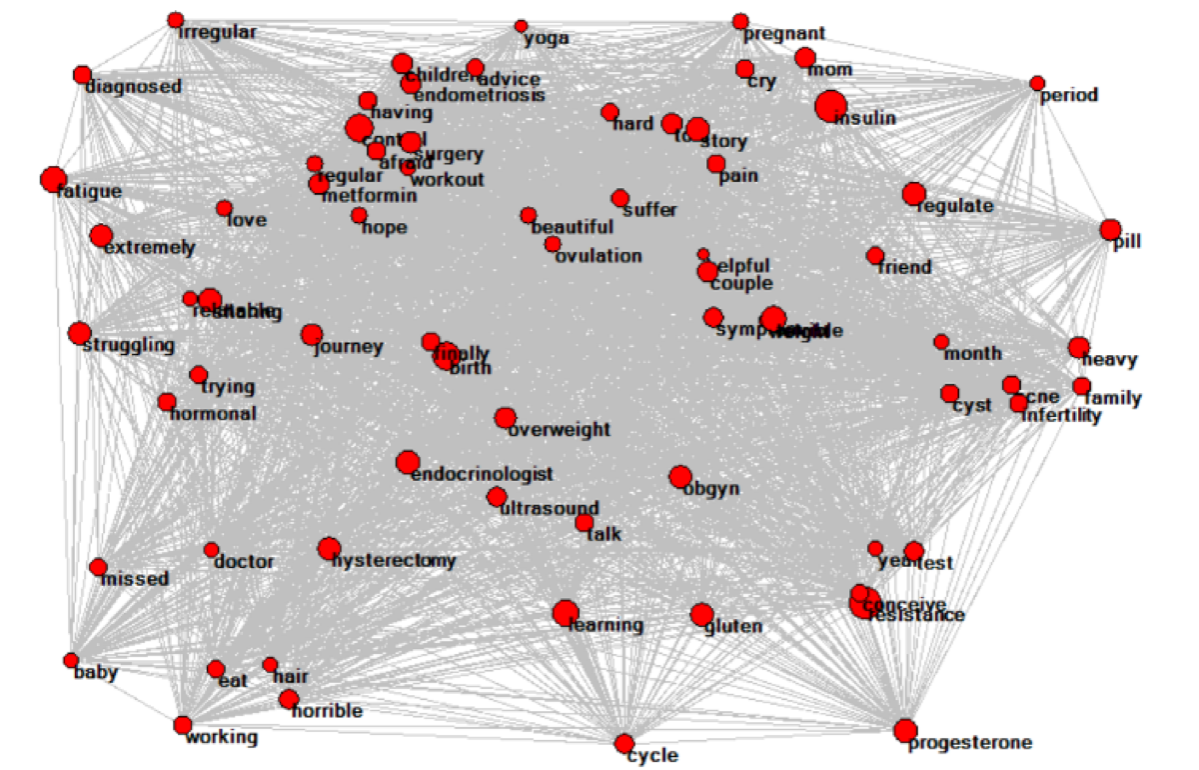
Network analysis of word association detection for keywords more frequently used by female commenters.

**Figure 3:**
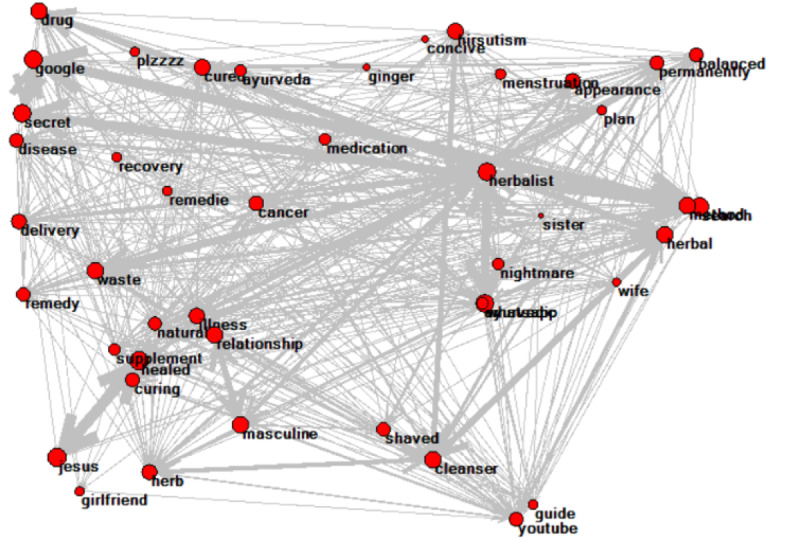
Network analysis of word association detection for keywords more frequently used by male commenters.

The various gender-specific keywords are listed in Table 1. Keywords associated with diagnosing PCOS, symptoms of PCOS, pills for PCOS (medication), and pregnancy were significantly associated with female users. Keywords associated with herbal treatment, natural treatment, curing PCOS, and online searches were significantly associated with male users. Network analysis showed 72 nodes with 4912 connections (indicated by arrows) for female users (Figure 2), and 45 nodes with 996 connections for male users (Figure 3). The key themes associated with female users were symptoms of PCOS (such as *irregular periods* and *acne*), positive personal experiences (such as *helpful* and *love*), negative personal experiences (such as *fatigue* and *pain*), motherhood (such as *infertility* and *trying to conceive*), self-diagnosis, and use of professional terminology detailing their journey (Table 2). The key themes associated with male users were misinformation regarding the “cure” for PCOS; using natural and herbal remedies to cure PCOS; fake or spam testimonies in which spammers disguised themselves as patients to sell their courses and consultations; finding treatment for PCOS; sharing perspectives of female family members, such as their partners; or relationship experiences (Table 3).

**Table 1:** Statistically significant keywords used by men and women in comparison with each other. *P* values represent the Benjamini-Hochberg significance.

Keyword			Chi-square (*DiffInP z* for *<M>–<F>*)
**Statistically significant keywords used by men compared to women**			
	Herbal	90.9 (9.5)***	
	Herb	26.9 (5.2)***	
	Natural	20.3 (4.5)*	
	Cleanser	24 (4.9**	
	Cured	59.4 (7.7)***	
	Curing	25.5 (5.0)**	
	Secret	61.7 (7.9)***	
	Girlfriend	33.7 (5.8)***	
	Wife	89.5 (9.5)***	
	Google	40 (6.3)***	
	YouTube	52.6 (7.3)***	
	Search	30.8 (5.6)***	
**Statistically significant keywords used by women compared to men**			
	Diagnosed	65.5 (–8.1)***	
	Symptoms	31 (–5.6)***	
	Periods	29.6 (–5.4)***	
	Weight	21.9 (–4.7)**	
	Birth	20 (–4.5)*	
	Endometriosis	19.4 (–4.4)*	
	Pill	19.2 (–4.4)*	
	Feel	18.8 (–4.3)*	

*P<.05.

**P<.01.

***P<.001.

**Table 2:** Content analysis of the keywords from YouTube comments used more frequently by female users with examples.

Broad theme	Subthemes	Examples (slightly paraphrased to prevent identification of users and ensure ease of reading)
Symptoms of PCOS^a^	Irregular periods, endometriosis, acne, heavy periods, infertility, cyst, missed periods, hair (hirsutism)	“I received a PCOS diagnosis approximately a year ago, and my physician prescribed a medication to alleviate the symptoms. While it does regulate my menstrual cycle, it doesn’t have a significant impact on issues like weight gain, excess hair growth, and acne.” “Undoubtedly, managing these aspects remains a challenging endeavor.” “I’ve been dealing with this syndrome since I turned 16. Now at 21, I appeared to be making progress after undergoing treatment involving hormone pills for several months, nearly a year. However, lately, the issues are resurfacing. In fact, I’m just about to leave for my medical appointment. It would be disheartening if I have to resort to hormones or any other medication once more. I’ve been adhering to a specific diet, and while I do feel improved, the symptoms of PCOS persist. :'/”
Negative personal experiences	Fatigue, struggling, cry, extremely, horrible, suffer, pain	“Women, avoid using birth control! My hormonal balance went completely haywire while on it. I experienced depression, exhaustion, and extreme mood swings – it was a dreadful experience for me.” “I appreciate you making this video. I received a PCOS diagnosis around a decade ago and have battled with weight, excessive hair growth, hormone-induced headaches, persistent fatigue, depression, anxiety, and sleep issues. I’ve managed to address a few symptoms and cope with the remaining challenges as effectively as possible.”
Positive personal experiences	love, beautiful, helpful	“Thank you very much, doctor. I’ve been dealing with PCOS for three years now, so this is incredibly beneficial for me.” “I made an effort to grasp the majority of the information discussed. The comments are also enlightening. This video appears to be highly informative. Subtitles would enhance the experience further. The pairing of PCOS and infertility is truly disheartening. Nevertheless, these home remedies do pique my interest. Your sharing is much appreciated.”
Motherhood	infertility, couple, pregnancy, hysterectomy, trying, conceive, family	“I’m currently on metformin for PCOS, and I’ve undergone tubal ligation. My periods are irregular, and I’m aiming to conceive. What steps can I take to improve my chances of getting pregnant?” “I experience occasional bleeding between periods and sometimes miss them altogether. Despite being told I couldn’t have kids, I have a 3-year-old son. There’s always a possibility. I’m eager for another child, but my PCOS has worsened, making it challenging this time. However, hope remains.”
Self-diagnosis	someone, tell, doctor, suggest, journey	“I’m 17 and have been experiencing irregular menstrual cycles. There was a three-month gap, and now my period has been ongoing for around three weeks. After the three-month gap, I had a regular period, but this current one has lasted a while. Could this possibly be a sign of a health issue? I’m hesitant about seeking medical help.” “I’m 18 years old, and I’ve noticed weight gain, acne, oily skin, and dark patches on my neck. My periods are irregular, but I’m uncertain about the exact condition I might be facing. Can anyone provide insight into what this could be? Is PCOS a possibility?”
Professional terminology	progesterone, obgyn, ovulation, resistance, yoga, insulin, pill, endocrinologist, ultrasound	“How to monitor ovulation in cases of PCOS.” “I experienced three months of non-stop menstrual bleeding. Last month, I underwent a sonography and was diagnosed with polycystic ovarian disease (without acne, weight gain, or abnormal hair growth). Currently, my hemoglobin level is at 5.6 (severe anemia), which required me to undergo a blood transfusion. Today, I finished receiving three units of blood transfusion.”

^a^PCOS: polycystic ovary syndrome.

**Table 3:** Content analysis of the keywords from YouTube comments used more frequently by male users with examples.

Broad theme	Subthemes	Examples (slightly paraphrased to prevent identification of users and ensure ease of reading)
Misinformation regarding the “cure” for PCOS^a^	cure, cured, curing, cleaner	While some people mentioned that there is no permanent cure for PCOS, most of the examples were either related to suggesting untested natural cures, buying courses, or contacting other people who will provide a cure. Direct examples are not listed here to prevent sharing identifying information.
Natural remedies	herb, herbal, herbalist, natural, Ayurveda, ginger	“Is it okay to consume orange tea and flaxseeds before bedtime? Also, if I incorporate all nine of these items, will it lead to a permanent cure for my PCOS, or could it recur? Nevertheless, I appreciate this video – it’s going to be beneficial for me.” “Find a cure for PCOS by strictly adhering to the Alkaline Diet!”
Fake/spam patient testimonies	search, YouTube, Google, WhatsApp, contact	Various commenters presented themselves as patients and spammed the same comments on multiple videos on how they had been affected by PCOS for multiple years and that when they contacted a spammer, the spammer miraculously treated their PCOS, and now they are living happily and also able to conceive. Then they provided all the contact details of the spammer. Direct examples are not listed here to prevent sharing identifying information.
Treatment	medication, supplement, recovery, remedies, secret	“I’m watching this because my soon-to-be wife has this condition, and I want to learn how to support her effectively. We’re currently exploring the use of supplements.” “My wife has been dealing with hirsutism, which results in unwanted hair growth. Even though I was understanding, it still affected her confidence negatively. To restore her happiness, I started researching solutions and got in touch with an herbal practitioner.”
Sharing perspectives of female family members	sister, girlfriend, wife, relationship	“Appreciate your input, doctor. My wife is going through a similar situation. Despite our efforts, we’ve been struggling to conceive. This is causing considerable anxiety and stress for me.” “This was a challenging period for me; my relationship was in jeopardy.”

^a^PCOS: polycystic ovary syndrome.

### Sentiment Analysis

**Figure 4:**
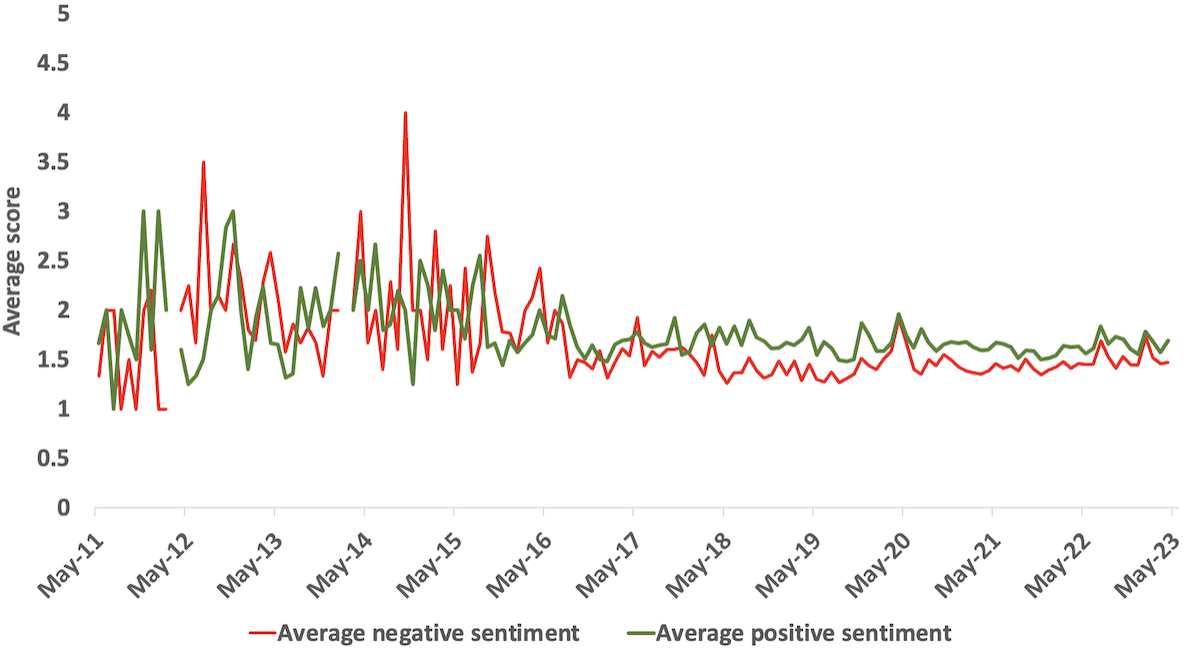
Sentiment trends of the YouTube comments overall from 2011 to 2023. There were no new comments in March 2012 or February 2014.

The average positive and negative sentiment scores associated with the extracted comments overall were 1.6651 (95% CI 1.6593-1.6709) and 1.4742 (95% CI 1.4683-1.4802), respectively, with a net positive difference of 0.1909. The average positive and negative sentiment scores for female users were 1.8266 (95% CI 1.8101-1.8430) and 1.6944 (95% CI 1.6750-1.7139), respectively, with a net positive difference of 0.1321. Male users’ average positive and negative sentiment scores were 1.7337 (95% CI 1.6892-1.7783) and 1.5279 (95% CI 1.4808-1.5750), respectively, with a net positive difference of 0.2058. The sentiments were majority positive when the estimated sentiment strength was weak or moderate (sentiment strength 2 or 3). However, negative sentiments dominated when the estimated sentiment strength was strong or very strong (sentiment strength 4 or 5; Table 4). Female users with very strong sentiment strength had higher negative sentiments when compared to similar male users. From 2011 to 2016, numerous variations in sentiments were seen. From 2017 onwards, the sentiment trends appear to have plateaued with slightly more positive sentiment (Figure 4).

**Table 4:** Sentiment analysis of the YouTube comments from 2011 to 2023 with gender distribution and overall trends.

	Any gender (N=85,872)		Male (n=1506)		Female (n=11,601)		Gender not identified (n=72,765)	
Score	Positive	Negative	Positive	Negative	Positive	Negative	Positive	Negative
1	57.12%	71.91%	52.92%	69.19%	47.39%	63.25%	58.76%	73.35%
2	21.77%	15.86%	23.31%	17.2%	26.14%	16.5%	21.04%	15.73%
3	18.66%	5.65%	21.51%	5.98%	23.07%	9.08%	17.9%	5.1%
4	2.35%	6.05%	1.99%	6.91%	3.23%	9.9%	2.22%	5.42%
5	0.09%	0.53%	0.27%	0.73%	0.17%	1.28%	0.07%	0.4%

## Discussion

### Principal Findings

To the best of our knowledge, this is the first attempt to comprehensively study all YouTube comments related to PCOS for evidence synthesis. While comments from female users revolved around lived experiences, those from male users focused on selling cures and spam or fake news. The sentiment trend has flattened and is slightly positive in the last few years, especially following May 2017, which may suggest an improved lived experience with PCOS in recent times, as seen graphically (Figure 4). Further studies are needed to study temporal trends in sentiment analysis, as our study yields weak evidence about slight positive sentiment. Positive stories highlight the importance of supporting networks and practical strategies in managing worries and discomfort within the context of PCOS conversations. Viewers express gratitude toward YouTubers who share their personal journeys and raise awareness about PCOS. Conversely, negative experiences shed light on the psychosocial distress accompanying this condition, demanding attention and understanding.

Brandolini’s law asserts that refuting incorrect information takes much more work than generating and disseminating it [30,31]. To address the imbalance between spreading falsehoods and dispelling them, this law stresses the significance of fact-checking and promoting trustworthy sources of information. Therefore, more concrete efforts are needed from YouTube, medical professionals, the public, and concerned local and international organizations to tackle misinformation and ensure that people are not misguided.

Online misinformation and fake news can significantly harm women with PCOS. False claims about PCOS’s causes, symptoms, and treatments spread rapidly through social media and websites, leading to confusion and delayed medical care. Unproven remedies promoted as cures can divert women from evidence-based management, worsening their condition and mental well-being. Misinformation can influence lifestyle choices, encouraging extreme diets and exercise routines that impact health negatively. Distrust in medical professionals may arise, preventing informed decisions and proper care. Stigmatization and isolation can result from perpetuated stereotypes. Additionally, misinformation can misguide family planning decisions due to misconceptions about fertility and pregnancy. Addressing this issue requires promoting digital literacy, reliable health resources, and supportive online communities to counterbalance the harmful effects of misinformation and ensure that women with PCOS receive accurate information and care.

Analyzing the sentiment of social media data from women with PCOS provides insights into their emotional well-being, highlighting the challenges, anxieties, and support-seeking behaviors they share online. By monitoring sentiment changes over time, health care providers can gauge the effectiveness of interventions and tailor support accordingly. It also improves patient-provider communication, allowing health care professionals to address specific concerns during appointments. Sentiment analysis informs targeted awareness campaigns that resonate with women’s emotional experiences, reducing stigma and promoting accurate information. Moreover, it sheds light on the psychological impact of PCOS, guiding research and interventions to address not only physical but also emotional aspects of the condition.

Several studies have highlighted gender disparities in diagnosis, management, and funding. And gender differences may exacerbate health disparities for health conditions that affect a single sex. Gender bias in health care has manifested through the underrepresentation of women in studies, trivialization of their complaints, and biased research grant awards [32]. Women’s exclusion from clinical trials like the aspirin study prompted action in the 1980s to include them, but issues persisted [33]. Women’s physical complaints historically were wrongly considered to be psychological, as seen with “hysteria.” Myalgic encephalomyelitis/chronic fatigue syndrome was initially dismissed as psychogenic [34]. Gender bias extends to research grants, with studies suggesting women face bias during grant renewals [35]. Canadian research grant analysis indicated biases in assessing female principal investigators based on their gender, not their research quality [36].

International awareness initiatives such as PCOS Awareness Month (September) may be used to develop targeted action-driven campaigning and involve underrepresented communities from low-, middle-, and high-income countries in fostering pragmatic communities. Dedicated themes may be used each year for a specific topic concerning PCOS as has been done and suggested for other awareness campaigns [37-39].

### Strengths and Limitations

The strengths of our study include the analysis of the comments on all PCOS videos on YouTube over 12 years. We used a multidimensional approach that included association mining, network analysis, subgroup analysis based on gender, sentiment analysis, and overall trends to analyze all the extracted YouTube comments. The methodology has been previously tested in various peer-reviewed studies. However, as commenting on YouTube videos is optional, an overrepresentation of participation by one gender depending on the topic may be seen (as in our case, since PCOS is a female-specific condition), limiting the generalizability of our findings. Gender estimation and sentiment assessment also have their limitations. Several usernames did not fit our classification criteria and were left unassigned. Although our methodology can distinguish between predominately male and female users, it is ineffective for nonbinary genders, possibly because people are named by their parents before they establish their gender identity; there are thus no nonbinary-specific first names [40]. The reliability of our findings may be affected by videos with clickbait titles or descriptions. Furthermore, the inherent methodological limitations of sentiment analysis, association mining, and network analysis cannot be disregarded.

It is important to note that social media should be viewed as one source among others when attempting to understand public views and sentiments. Its limitations include the potential for echo chambers, manipulation of information, varied dynamic trends corresponding to public events [41,42], and the exclusion of individuals who are not active on these platforms. Therefore, combining social media analysis with other research methods, such as surveys, focus groups, and traditional media analysis, can provide a more comprehensive understanding of public views and sentiments. Nonetheless, our study shares important coverage on the public dissemination of PCOS-related information.

### Conclusions

There has been an increasing trend for sharing information about PCOS on YouTube in recent years, with a similar rise in viewers sharing their perspectives. There is a disparity in views on PCOS between women and men, with the latter associated with non–evidence-based approaches and misinformation. The improving sentiment noticed with YouTube comments may reflect better health care services recently. Prioritizing and promoting evidence-based care and disseminating pragmatic online coverage is warranted to improve public sentiment and limit misinformation spread.

## Abbreviations

PCOS: polycystic ovary syndrome

## Data Availability

The data sets generated during and/or analyzed during this study are available from the corresponding author on reasonable request.

## References

[1] Joham AE, Norman RJ, Stener-Victorin E, Legro RS, Franks S, Moran LJ, Boyle J, Teede HJ (2022). Polycystic ovary syndrome. Lancet Diabetes Endocrinol.

[2] Tay C, Garrad Rhonda, Mousa A, Bahri Mahnaz, Joham A, Teede Helena (2023). Polycystic ovary syndrome (PCOS): international collaboration to translate evidence and guide future research. J Endocrinol.

[3] Lau GM, Elghobashy M, Thanki M, Ibegbulam S, Latthe P, Gillett CDT, O'Reilly Michael W, Arlt W, Lindenmeyer A, Kempegowda P, PCOS SEva Working Group (2022). A systematic review of lived experiences of people with polycystic ovary syndrome highlights the need for holistic care and co-creation of educational resources. Front Endocrinol (Lausanne).

[4] Saei Ghare Naz M, Ramezani Tehrani Fahimeh, Ozgoli G (2019). Polycystic Ovary Syndrome in adolescents: a qualitative study. Psychol Res Behav Manag.

[5] Ismayilova M, Yaya Sanni (2023). 'I'm usually being my own doctor': women's experiences of managing polycystic ovary syndrome in Canada. Int Health.

[6] Hadjiconstantinou M, Mani H, Patel N, Levy M, Davies M, Khunti K, Stone Margaret (2017). Understanding and supporting women with polycystic ovary syndrome: a qualitative study in an ethnically diverse UK sample. Endocr Connect.

[7] Sheikh J, Khalil H, Shaikh S, Hebbar M, Zia N, Wicks S, Jayaprakash S, Narendran A, Subramanian A, Malhotra K, Chapman R, Gillett C, Gleeson HK, Robinson L, Chu JJ, Lathia T, Selvan C, O'Reilly MW, Manolopoulos KN, Arlt W, Kempegowda P, PCOS SEva team (2023). Emotional and psychosexual well-being is influenced by ethnicity and birthplace in women and individuals with polycystic ovary syndrome in the UK and India. BJOG.

[8] Eysenbach G (2009). Infodemiology and infoveillance: framework for an emerging set of public health informatics methods to analyze search, communication and publication behavior on the Internet. J Med Internet Res.

[9] Mallappallil M, Sabu J, Gruessner A, Salifu M (2020). A review of big data and medical research. SAGE Open Med.

[10] Ristevski B, Chen Ming (2018). Big data analytics in medicine and healthcare. J Integr Bioinform.

[11] Borges do Nascimento IJ, Pizarro Ana Beatriz, Almeida J, Azzopardi-Muscat N, Gonçalves Marcos André, Björklund Maria, Novillo-Ortiz D (2022). Infodemics and health misinformation: a systematic review of reviews. Bull World Health Organ.

[12] Malhotra K, Pan CSC, Davitadze M, Kempegowda Punith, Team PCOS SEva (2023). Identifying the challenges and opportunities of PCOS awareness month by analysing its global digital impact. Front Endocrinol (Lausanne).

[13] Elhariry M, Malhotra K, Solomon M, Goyal K, Kempegowda P (2022). Top 100 #PCOS influencers: Understanding who, why and how online content for PCOS is influenced. Front Endocrinol (Lausanne).

[14] Ozduran E, Büyükçoban Sibel (2022). A content analysis of the reliability and quality of Youtube videos as a source of information on health-related post-COVID pain. PeerJ.

[15] Memioglu T, Ozyasar M (2022). Analysis of YouTube videos as a source of information for myocarditis during the COVID-19 pandemic. Clin Res Cardiol.

[16] Jia X, Pang Y, Liu LS (2021). Online health information seeking behavior: A systematic review. Healthcare (Basel).

[17] Andan C, Aydin Mustafa F (2022). Evaluation of the reliability and quality of YouTube videos on ovarian cysts. Cureus.

[18] Atigan A, Atigan Alev (2023). Polycystic ovary syndrome and exercise: evaluation of YouTube videos. Cureus.

[19] Thelwall M, Mas-Bleda A (2018). YouTube science channel video presenters and comments: female friendly or vestiges of sexism?. Aslib J Inf Manag.

[20] Thelwall M Social web text analytics with Mozdeh.

[21] Benjamini Y, Hochberg Y (2018). Controlling the false discovery rate: A practical and powerful approach to multiple testing. J R Stat Soc Series B Stat Methodol.

[22] CIShell manual : Fruchterman-Reingold with annotation (prefuse beta). CIShell.

[23] Hansen D, Shneiderman B, Smith M, Himelboim I (2020). Installation, orientation, and layout. Analyzing Social Media Networks with NodeXL.

[24] Larivière Vincent, Ni C, Gingras Y, Cronin B, Sugimoto CR (2013). Bibliometrics: global gender disparities in science. Nature.

[25] Thelwall M, Buckley K, Paltoglou G (2011). Sentiment strength detection for the social web. J Am Soc Inf Sci.

[26] Taboada M, Brooke J, Tofiloski M, Voll K, Stede M (2011). Lexicon-based methods for sentiment analysis. Comput Linguist.

[27] Pang B, Lee L (2008). Opinion mining and sentiment analysis. Found Trends Inf Retr.

[28] Thelwall M, Cash S (2021). Bullying discussions in UK female influencers’ YouTube comments. Br J Guid Couns.

[29] Schenker N, Gentleman Jf (2001). On judging the significance of differences by examining the overlap between confidence intervals. Am Stat.

[30] Dijkstra S, Kok G, Ledford JG, Sandalova E, Stevelink R (2018). Possibilities and pitfalls of social media for translational medicine. Front Med (Lausanne).

[31] Thatcher J, Shears A, Eckert J (2018). Thinking Big Data in Geography: New Regimes, New Research.

[32] Mirin AA (2021). Gender disparity in the funding of diseases by the U.S. National Institutes of Health. J Womens Health (Larchmt).

[33] Steering Committee of the Physicians' Health Study Research Group (1989). Final report on the aspirin component of the ongoing Physicians' Health Study. N Engl J Med.

[34] Mirin AA, Dimmock ME, Jason LA (2020). Research update: The relation between ME/CFS disease burden and research funding in the USA. Work.

[35] Kaatz A, Lee Y, Potvien A, Magua Wairimu, Filut Amarette, Bhattacharya Anupama, Leatherberry Renee, Zhu Xiaojin, Carnes Molly (2016). Analysis of National Institutes of Health R01 application critiques, impact, and criteria scores: does the sex of the principal investigator make a difference?. Acad Med.

[36] Witteman HO, Hendricks M, Straus S, Tannenbaum C (2019). Are gender gaps due to evaluations of the applicant or the science? A natural experiment at a national funding agency. Lancet.

[37] Malhotra K, Kalra A, Kumar A, Majmundar M, Wander G, Bawa Ashvind (2022). Understanding the digital impact of World Hypertension Day: key takeaways. Eur Heart J Digit Health.

[38] Malhotra K, Bawa A, Goyal K, Wander GS (2022). Global impact of deep vein thrombosis awareness month: challenges and future recommendations. Eur Heart J.

[39] Goyal K, Nafri A, Marwah M, Aramadaka Saikumar, Aggarwal Pranshul, Malhotra Sakshi, Mannam Raam, Gupta Oman, Malhotra Kashish (2022). Evaluating the global impact of Stroke Awareness Month: a serial cross-sectional analysis. Cureus.

[40] Thelwall M, Thelwall S, Fairclough R (2021). Male, female, and nonbinary differences in UK Twitter self-descriptions: a fine-grained systematic exploration. J Data Inf Sci.

[41] Park HC, Youn JM, Park HW (2018). Global mapping of scientific information exchange using altmetric data. Qual Quant.

[42] Jalali S, Park H, Vanani I, Pho KH (2021). Research trends on big data domain using text mining algorithms. Digit Scholarsh Humanit.

